# Workers’ Memorial Day — April 28, 2015

**Published:** 2015-04-24

Workers’ Memorial Day, observed each year on April 28, recognizes workers who died or who have experienced exposures to hazards at work. In 2013, a total of 4,405 U.S. workers died from work-related injuries (1); in 2007, according to the latest estimate available, 53,445 deaths could be attributed to work-related illness (2).

In 2013, approximately 3 million injuries and illnesses to private industry workers and 746,000 to state and local government workers were reported by employers (3). In the same year, an estimated 2.8 million work-related injuries were treated in emergency departments, resulting in 140,000 hospitalizations (National Institute for Occupational Safety and Health, unpublished data, 2014).

Although certain national surveillance systems (4) record new cases of selected nonfatal work-related illnesses, the overall incidence of such illness is not well documented. This issue of *MMWR* includes a report on work-related asthma, one of many under-recognized work-related illnesses, in addition to a report on occupational traumatic injuries among health care workers. In 2007, the cost of work-related fatalities, injuries, and illnesses in the United States was estimated at $250 billion (2). CDC is working to better describe the overall societal burden of occupational fatalities, injuries, and illnesses; additional information is available at http://www.cdc.gov/niosh/programs/econ/risks.html.
